# Evaluating the Effectiveness of the Health Management Program for the Elderly on Health-Related Quality of Life among Elderly People in China: Findings from the China Health and Retirement Longitudinal Study

**DOI:** 10.3390/ijerph16010113

**Published:** 2019-01-03

**Authors:** Xiuqi Hao, Yuehan Yang, Xiaotong Gao, Tao Dai

**Affiliations:** 1 Peking Union Medical College, Beijing 100730, China; haoxiuqi1992@foxmail.com (X.H.); gaoxiaotong1994@foxmail.com (X.G.); 2 Institute of Medical Information & Library, Chinese Academy of Medical Sciences, Beijing 100020, China; yang.yuehan@imicams.ac.cn

**Keywords:** elderly, health-related quality of life, effectiveness, health management

## Abstract

The world’s rapidly aging population brings serious challenges which could be addressed by changes in behaviour and policy that promote good health in older age. However, these cheap and simple interventions are not available in many countries. China is one of the fastest-ageing countries in the world. The health management programs for the elderly in basic public health services was introduced by the government to promote the health of the elderly in China and address the challenges related to ageing. However, the effectiveness of the program is uncertain. So, we use a propensity score matching difference-in-difference (PSM-DID) model to analyse the causal effect of the health management program for the elderly in basic public health services on the health-related quality of life (HRQoL) of the elderly in China. The result shows that the program has improved the physical health of the elderly but has had no significant impact on mental health. Expanding the program to cover mental health could further benefit the HRQoL of the elderly. The program is a cost-effective approach to tackle the challenges of ageing and is a good example for other developing countries facing the same ageing challenges.

## 1. Introduction

According to the World Population Prospects, the world’s population is rapidly ageing: in 2018, the number of people over 65 years of age in the world will exceed the number of children under 5 years of age for the first time in history [1]. Population ageing is strongly related to rises in the prevalence of chronic conditions and disability, such as diabetes, stroke, chronic obstructive pulmonary disease and unintentional injury [2,3]. It poses a serious challenge to the world, but evidence suggests that this challenge can be addressed by changes in behaviour and policy, especially those that promote good health in older age such as the effective management of common chronic diseases and the promotion of healthy lifestyles [4]. However, in most countries these cheap and simple interventions are not available [5].

China has the largest number of elderly people (65 years and older) in the world, reaching 150 million, and it is one of the fastest-ageing countries [6]. The challenges facing China are even more daunting than those elsewhere. China’s rapidly ageing population is expected to be associated with at least a 40% increase in the burden of chronic non-communicable diseases by the year 2030 [7]. To address this challenge, the Chinese government included health management programs for the elderly in the Basic Public Health Services (BPHS) proposed in the new round of health reform launched in 2009 [8]. The health management program for the elderly is a government-subsidized comprehensive health management service for all community residents aged 65 and older, provided by primary health-care institutions once a year, and the package includes lifestyle and health assessment, physical examination, auxiliary examination and health education based on the results of the examination. It intends to promote the health of the elderly by detecting and treating disease or injury at an early stage, using personalised health education to prevent the occurrence and development of disease and to reduce complications and disability. In 2015, the program covered 118 million people over the age of 65 in China [9]. Wang [10] assessed the difference before and after the implementation of the health management program for the elderly with the chi-square test, and found that lifestyle and behaviour related to chronic conditions and the awareness rate and treatment rate of hypertension and diabetes mellitus significantly improved, but the evidence is limited by the nature of uncontrolled before–after studies. The effectiveness of the program is still uncertain.

The aim of this study is to evaluate the effectiveness of the health management program for the elderly in basic public health services on the health-related quality of life among elderly people in China and to provide evidence for health policy-makers and researchers to design or improve public health intervention strategies for elderly people. First, the study developed and validated a new scale to measure health-related quality of life, and we then used data from the China Health and Retirement Longitudinal Study and the propensity score matching difference-in-difference (PSM-DID) model to analyse the causal effect of the program on the HRQoL of the elderly in China.

## 2. Materials and Methods

### 2.1. Data Sources and Sampling

Data were obtained from the China Health and Retirement Longitudinal Study (CHARLS, Beijing, China) 2011–2015 waves [11]. CHARLS is a nationally representative, publicly available microdatabase based on a sample of households with members aged 45 years or above. The baseline national wave of CHARLS was conducted between June 2011 and March 2012 by the Institute of Social Science Survey, Peking University using multistage, probability proportional to size sampling stratified by regions and then by urban districts/rural counties and per capita GDP. It covered 17,705 respondents from 10,257 households among 150 counties/districts in 28 provinces. The interview used a face-to-face computer-assisted personal interview approach by well-trained interviewers. The CHARLS sample is very similar to the Chinese national population according to the Chinese population census of 2010. Detailed information about the CHARLS can be found at http://charls.pku.edu.cn/en.

In this study, we used the CHARLS data from the 2011 wave and the 2015 wave to create a panel dataset that includes elderly people who completed the follow-up. Because the health management program for the elderly is provided for all community residents aged 65 and older, and to construct a baseline of those who did not receive the intervention to apply PSM-DID, the present study only includes people born before 1946 who were necessarily 65 years of age or older and who had not received the health management program for the elderly in the 2011 baseline survey. The health management programs for the elderly provide a government-subsidized physical examination once a year for all community residents aged ≥65 years, and the government also provides a free physical examination at least once a year for those community residents aged ≥35 years who have been diagnosed with hypertension or diabetes and people with severe mental illness living in communities. From the samples we removed individuals who had been diagnosed with hypertension, diabetes or mental illness, so that in this study the health management program for the elderly could be identified by whether an individual received a government-subsidized physical examination. We constructed a treatment variable using the variables “ec001_1” and “ec002” from CHARLS. We used the variable “ec001_1: When did you take the last physical examination?” to identify persons who had taken a physical examination in the past two years because CHARLS data were collected every two years. Then, we used the variable “ec002: Who paid the physical examination cost?” and the answer “government” to identify persons who received a government-subsidized physical examination. The treatment group consisted of people who received the health management program for the elderly in the 2015 wave, and the control group included those who did not receive the health management program for the elderly in the 2011 wave or in the 2015 wave of CHARLS.

### 2.2. Measures

To measure the health-related quality of life of the elderly, we developed and validated a new scale based on the Short Form 36 (SF-36) and the CHARLS variables. The SF-36 is the most frequently used self-administered screening tool for health-related quality of life; it is a 36-question comprehensive health survey that assesses eight health concepts used to define quality of life [12]. The CHARLS questionnaire itself does not contain the SF-36, but the CHARLS questionnaire contains a wide range of health indicators including activities of daily living (ADLs), instrumental activities of daily living (IADLs) and measures of physical activities and physical functioning, as well as questions on mental health (depression), cognitive capability and subjective expectations of living to certain future ages. The constructed SF-36 based on the CHARLS variables selected and adapted questionnaire items that measure eight subscales of the original SF-36 [12] (see Table 1 and supplementary materials). Six experts with a public health background were invited to discuss the selection of CHARLS variables based on the original SF-36 scale, as well as a content validity evaluation. We recoded the values for CHARLS variables such that a higher-scored item value indicates a poor health state. The scores of the eight scales of SF-36—physical functioning, role—physical, bodily pain, general health, vitality, social functioning, role—emotional, and mental health—were computed by summing the item scores and then transforming the raw score to a 0-to-100 scale. The scores of the eight subscales were summarised into two aggregate scores according to the conceptual model of SF-36 [13]. The physical component score (PCS) for physical functioning, physical role, bodily pain, and general health perception and the mental component score (MCS) for mental health, vitality, emotional role, and social functioning were computed.

The reliability of the constructed SF-36 based on the CHARLS variables was measured by Cronbach’s α coefficient. Except for Vitality (α = 0.34) and Role—emotional (α = 0.60), each dimension of the constructed SF-36 based on the CHARLS variables has an alpha value greater than 0.7, which is an acceptable value indicating good internal consistency. For content validity, experts were invited to discuss the design and content of the constructed SF-36 based on the CHARLS variables. A content validity index was calculated at both the item level (I-CVI) and scale level (S-CVI). An I-CVI of 0.78 or higher and an S-CVI above 0.9 are considered excellent. The I-CVIs ranged from 0.83 to 1.00 and the Average CVI (S-CVI/Ave) was 0.94, indicating excellent content validity. Our findings support the reliability and validity of the constructed SF-36 based on the CHARLS variables for assessing health status among elderly people in China.

### 2.3. Data Analysis

We used the propensity score matching difference-in-difference (PSM-DID) model proposed by Heckman, which is widely used in health policy evaluations [14]. Difference-in-difference models compare changes over time in the control group to changes over time in the treatment group and attribute the DID estimator to the effect of the policy. Difference-in-difference methods provide unbiased effect estimates if the trend over time would have been the same between the treatment and control groups in the absence of the intervention. However, in this study, the health management program for the elderly is not mandatory and the participants may self-select into treatment; this may cause participants and nonparticipants to be incomparable, so there may have been different trends over time. We used a propensity score matching method to match people in the treatment group with people from the control group with the closest propensity score, making the observed characteristics of the two groups comparable and reducing selection bias.

In our study, there were more people in the control group than in the intervention group, so we chose the kernel matching method which uses a weighted average of all participants in the control group to construct a counterfactual outcome. Therefore, a major advantage of the methods in our study is the lower variance obtained due to the use of more information. The disadvantage of this method is that the observations that are used may be bad matches. Therefore, it is important that the common support assumption is satisfied for kernel matching so that only persons with suitable control cases are considered. We checked the overlap and the region of common support between the treatment group and the control group using a visual analysis of the density distribution of the propensity score in both groups, as suggested by many previous studies [15].

The propensity score represents the probability of participating in the intervention, conditional on the observable characteristics. Since we condition not on all covariates but on the one-dimensional propensity score, we checked whether the matching procedure can balance the distribution of the relevant covariates in both the treatment and control groups using the method recommended by Rubin [16], such as a two-sample *t*-test and standardised bias.

We used a rich set of control variables that were measured before the treatment, including gender, age, marital status, educational level, number of children, medical insurance, household income per capita, smoking history, alcohol consumption history, nighttime sleep time and number of meals per day.

PSM was used to eliminate as much observed heterogeneity as possible. Then, it was combined with a difference-in-difference (DID) model to remove unobserved fixed effects through within-person comparisons over time as well as the common period and ageing effects by comparing the trends of treatment and control groups to evaluate the net effect of the health management program for the elderly on the health-related quality of life of the elderly in China.

## 3. Results

**Figure 1:**
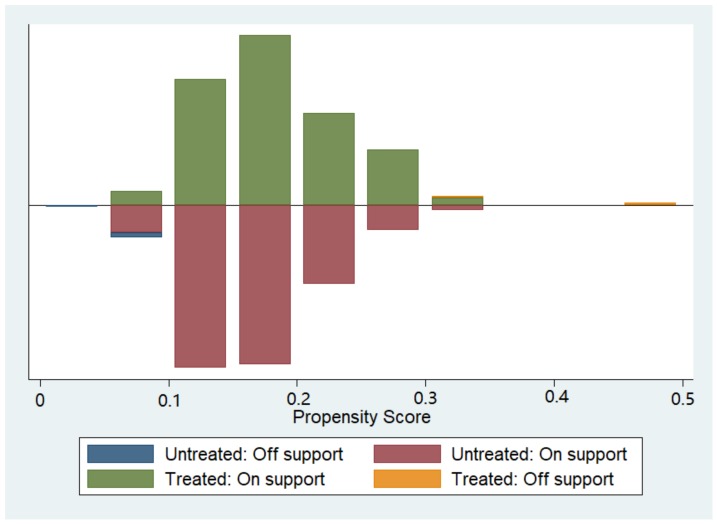
Visual analysis of the validity of the common support assumption.

A total of 3646 elderly people aged 65 and older were followed up in the 2015 wave of CHARLS. After excluding patients diagnosed with hypertension, diabetes or mental illness and people who received the health management program in 2011 or had missing values in the main variables, 1211 elderly people were included in the study. Figure 1 illustrates a visual analysis of the validity of the common support assumption by the distribution of the propensity scores for the treatment group and the control group. The treatment group is shown above the midline, and the control group is shown below the midline; the figure shows a large overlap in the propensity score between the two groups and the distribution is very similar, thus confirming the validity of the common support assumption.

Table 2 compares the demographic characteristics of the treatment group and the control group before and after kernel propensity score matching.

Compared with the control group before matching, the treatment group was slightly younger (mean age 70.02 vs. 70.76), more likely to have a formal education (53.92% vs. 44.29%) and more likely to have a smoking history (52.94% vs. 44.69%) and alcohol consumption history (40.20% vs. 30.69%); the p-values of these differences were all less than 0.05. After matching, the differences in all covariates between the treatment group and the control group were not significant, and the standardised percentage bias was reduced significantly, indicating that the propensity score matching substantially reduced the between-group differences in observed characteristics.

As shown in Table 3, in 2011, the differences in all SF-36 subscale scores (except vitality) between the treatment group and the control group were statistically insignificant. In 2015, the scale scores of physical functioning of both groups had absolute reductions; there was a smaller reduction among the treatment group, and the difference attributable to the treatment was only statistically significant at the 10% level (3.00, SE = 1.67, *p* = 0.073). The scale scores of role—physical, bodily pain, and PCS of the treatment group were increased, while the same scores of the control group decreased in 2015; the difference attributable to the treatment was statistically significant at the 1% level (3.82, SE = 1.23, *p* = 0.002; 6.78, SE = 2.50, *p* = 0.006; 3.93, SE = 1.35, *p* = 0.004, respectively). For the scale scores of vitality, social functioning, role—emotional, mental health and MCS, the difference attributable to the treatment was statistically insignificant.

## 4. Discussion

The results showed that the health management program for the elderly significantly improved the scale scores of physical functioning, role—physical, and bodily pain scales and the PCS with a moderate effect size considering the ageing effect. As described in numerous studies [17], those four scales have been shown to be the most valid SF-36 scales for measuring physical health. The health management program for the elderly in basic public health services promotes the physical health of the elderly, which is consistent with the results of previous similar studies. A systematic review and meta-analysis of effectiveness of community-based interventions for elderly people reported that physical function was better after complex interventions for elderly people [18]. The success factors in our study could be the result of a multidimensional approach to detect and treat disease or injury at an early stage, and the findings of Andreas confirm the concept that functional status decline can be delayed or prevented by periodic health assessment for detection of modifiable risk factors and subsequent intervention to modify these risk factors and to identify new risks [19].

Although the program significantly improved the physical health of elderly people, it did not improve the general health score; this contradictory result may explained by the findings of a study by Behma [20], who found that a postponed progression of morbidity may not affect the self-rated health of the elderly, and there might be differences between how the elderly perceive their health and how satisfied they are with it. Also, under the stereotypic view that ageing is accompanied by illness, the elderly may be satisfied with their general health while experiencing deterioration in health.

We find that the program did not improve mental health measures such as mental health, role—emotional, vitality, and social functioning—which is in line with our assumptions since the program only includes interventions that focus on the physical aspect of health. A previous study [21] indicated that psychological wellbeing might be even more closely related to a high quality of life than good physical health and favorable socioeconomic circumstances. Despite the well-recognised direct links between mental health and health-related quality of life, health promotion programs for the elderly usually focus more on physical as opposed to mental health [22]. There are no routine mental health screenings for the elderly in the basic public health services. Mental health conditions are the fifth-leading cause of DALYs in the elderly people of China [23], but the progress of the primary health-care system in focusing on mental health remains slow in China [24]. Thus, we believe that the health management program for the elderly could be considerably improved by adding mental health services to the program, offering a promising route to improving the health-related quality of life of the elderly.

The basic public health services cost between $2.14 and $7.98 per capita, and the health management program for the elderly is only one of 12 programs in place in 2018, but it has caused a measurable improvement in physical health for the elderly. The health management program for the elderly in basic public health services might be a cost-effective approach to tackle ageing challenges and is a good example for other developing countries facing the same challenges.

Our study has limitations. Ageing is usually accompanied by cognitive decline, so some participants may not recall the exact time when they had a physical examination or who paid for the physical examination, and this may cause misclassification bias. Selection bias could be caused by missing values in the main variables or by loss to follow-up. Those biases can lead to overestimating or underestimating the intervention effect.

## 5. Conclusions

The health management program for the elderly in basic public health services in China has improved the physical health of the elderly but has had no significant impact on mental health. We suggest that the addition of mental health services to the health management program for the elderly is likely to contribute to a significant improvement in health-related quality of life among elderly people. The health management program for the elderly is a cost-effective approach to tackle ageing challenges and is a good example for other developing countries facing the same ageing challenges.

## Figures and Tables

**Table 1:** Selection of CHARLS variables based on the original Short Form 36 (SF-36) scale.

SF-36 Scales	CHARLS Variables
Physical functioning	Run1Km(DB001) Walk1km(DB002) Walk100m(DB003) DifficultyGetupchair(DB004) DifficultyClimbstairs(DB005) DifficultyKneeling(DB006) DifficultyExtendArms(DB007) DifficultyLift(DB008) DifficultyPickupCoin(DB009)
Role—physical	DifficultyHouseholdChores(DB016) DifficultyPrepareMeals(DB017) DifficultyShopping(DB018) DifficultyTakeMedications(DB020)
Bodily pain	AnyBodyPains(DA041) WhatPartBodyPain(DA042)
General health	SelfRatedHealth1(DA001) SelfRatedHealth2(DA002)
Vitality	SleepRestless(DC015) CouldNotGetGoing(DC018)
Social functioning	AnySocialActivities(DA056) FrequencyOfActivity(DA057)
Role—emotional	TroubleConcentrate(DC010) FeelHard(DC012)
Mental health	UnusuallyBothered(DC009) FeltDepressed(DC011) FeltHopeful(DC013) FeltFearful(DC014) Happy(DC016) FeltLonely(DC017)

Source: China Health and Retirement Longitudinal Study (CHARLS) (2011, 2015).

**Table 2:** Balancing test: demographic characteristics of the treatment group and the control group before and after matching.

Characteristic	Matching	Treatment	Control	*p*	%Bias	%Reduction Bias
Age (years)	Unmatched	70.02	70.76	0.04	−16.3	
	Matched	70.03	70.13	0.80	−2.4	85.5
Formal education (%)	Unmatched	53.92	44.29	0.01	19.3	
	Matched	53.47	50.63	0.57	5.7	70.5
Married (%)	Unmatched	79.90	79.74	0.96	0.4	
	Matched	80.20	80.15	0.99	0.1	67.3
Male (%)	Unmatched	60.78	54.72	0.11	12.3	
	Matched	60.89	58.49	0.62	4.9	60.4
Medical Insurance (%)	Unmatched	96.57	94.24	0.18	11.1	
	Matched	96.54	96.63	0.96	−0.5	95.9
Nighttime sleep time (hour)	Unmatched	6.04	6.09	0.73	−2.7	
	Matched	6.06	6.06	0.98	0.2	90.7
More than 4 meals per day (%)	Unmatched	1.96	1.09	0.31	7.1	
	Matched	0.99	1.5	0.65	−4.2	41.3
Smoking history (%)	Unmatched	52.94	44.69	0.03	16.5	
	Matched	52.97	50.14	0.57	5.7	65.8
Alcohol consumption history (%)	Unmatched	40.20	30.69	0.01	20	
	Matched	40.10	36.72	0.49	7.1	64.4
More than 2 children (%)	Unmatched	80.39	78.65	0.58	4.3	
	Matched	80.20	79.80	0.92	1	77.2
Household income per capita (yuan)	Unmatched	9088.10	12,237.00	0.23	−11.3	
	Matched	9534.00	8965.50	0.66	2	81.9

Source: CHARLS (2011). %Bias denotes the standardised percentage bias.

**Table 3:** Overall effect of the health management program for the elderly on the health-related quality of life of the elderly in China.

Outcome	Time	Treatment	Control	Difference	PSM-DID Estimator
Physical functioning	2011	82.19	81.29	0.91 (1.18)	
	2015	78.94	75.03	3.91 *** (1.18)	3.00 * (1.67)
Role—physical	2011	94.80	94.91	−0.11 (0.87)	
	2015	95.05	91.34	3.71 *** (0.87)	3.82 *** (1.23)
Bodily pain	2011	79.74	80.22	−0.48 (1.76)	
	2015	84.14	77.83	6.31 *** (1.76)	6.78 *** (2.50)
General health	2011	39.60	37.36	2.25 (1.48)	
	2015	40.72	36.36	4.36 *** (1.48)	2.11 (2.10)
Vitality	2011	77.72	75.04	2.69 * (1.56)	
	2015	80.86	74.90	5.96 *** (1.56)	3.27 (2.21)
Social functioning	2011	27.89	26.47	1.41 (1.70)	
	2015	28.11	26.98	1.12 (1.70)	−0.29 (2.41)
Role—emotional	2011	66.09	66.66	−0.57 (1.84)	
	2015	73.02	70.42	2.60 (1.84)	3.169 (2.60)
Mental health	2011	71.40	71.21	0.19 (1.27)	
	2015	74.49	71.38	3.11 ** (1.27)	2.92 (1.80)
PCS	2011	74.09	73.44	0.64 (0.96)	
	2015	74.71	70.14	4.57 *** (0.96)	3.93 *** (1.35)
MCS	2011	60.78	59.85	0.93 (1.07)	
	2015	64.12	60.92	3.19 *** (1.07)	2.27 (1.52)

Source: CHARLS (2011, 2015). Standard errors in parentheses, *** *p* < 0.01, ** *p* < 0.05, * *p* < 0.1.

## Supplementary Materials

The following are available online at http://www.mdpi.com/1660-4601/16/1/113/s1, Figure S1: title, Table S1: title, Video S1: title.
