# Supplementary material for: Evaluating the Effectiveness of the Health Management Program for the Elderly on Health-Related Quality of Life among Elderly People in China: Findings from the China Health and Retirement Longitudinal Study
Source: Int J Environ Res Public Health. 2019 Jan 3;16(1):113. doi: 10.3390/ijerph16010113 (PMC6338948; doi:10.3390/ijerph16010113)
Supplement: Supplementary file 1 [file ijerph-16-00113-s001.pdf]

Detailed information of selected CHARLS variables of the new scale

Physical functioning

Run1Km(DB001) Do you have any difficulty with running or jogging about 1 Km?

1. No, I don’t have any difficulty.
2. I have difficulty but can still do it.
3. Yes, I have difficulty and need help.
4. I can not do it.

Walk1km(DB002) Do you have difficulty …Walking 1 km…?

1. No, I don’t have any difficulty.
2. I have difficulty but can still do it.
3. Yes, I have difficulty and need help.
4. I can not do it.

Walk100m(DB003) Do you have difficulty …Walking 100 metres…?

1. No, I don’t have any difficulty.
2. I have difficulty but can still do it.
3. Yes, I have difficulty and need help.
4. I can not do it.

DifficultyGetupchair(DB004) Do you have difficulty …Getting up from a chair after sitting for a long period…?

1. No, I don’t have any difficulty.
2. I have difficulty but can still do it.
3. Yes, I have difficulty and need help.
4. I can not do it.

DifficultyClimbstairs(DB005) Do you have difficulty …Climbing several flights of stairs without resting…?

1. No, I don’t have any difficulty.
2. I have difficulty but can still do it.
3. Yes, I have difficulty and need help.
4. I can not do it.

DifficultyKneeling(DB006) Do you have difficulty …Stooping, kneeling, or crouching…?

1. No, I don’t have any difficulty.
2. I have difficulty but can still do it.
3. Yes, I have difficulty and need help.
4. I can not do it.

DifficultyExtendArms(DB007) Do you have difficulty …Reaching or extending your arms above shoulder level…?

(he/she is regarded as not having difficulty only if he/she can extend both of his/her arms, otherwise he/she is regarded as having difficulty.)

1. No, I don’t have any difficulty.
2. I have difficulty but can still do it.
3. Yes, I have difficulty and need help.
4. I can not do it.

DifficultyLift(DB008) Do you have difficulty …Lifting or carrying weights over 10 jin, like a heavy bag of groceries…?
1. No, I don't have any difficulty.
2. I have difficulty but can still do it.
3. Yes, I have difficulty and need help.
4. I can not do it.
DifficultyPickupCoin(DB009) Do you have difficulty …Picking up a small coin from a table…?
1. No, I don't have any difficulty.
2. I have difficulty but can still do it.
3. Yes, I have difficulty and need help.
4. I can not do it.

Role-physical

DifficultyHouseholdChores(DB016) Because of health and memory problems, do you have any difficulties with doing household chores? (Definition: By doing household chores, we mean house cleaning, doing dishes, making the bed, and arranging the house.) [IWER: If R cannot mop the floor, but can scrub, or R cannot fold heavy bedding,but is able to do light ones, then mark (3).]
1. No, I don't have any difficulty
2. I have difficulty but can still do it.
3. Yes, I have difficulty and need help.
4. I can not do it.
DifficultyPrepareMeals(DB017) Because of health and memory problems, do you have any difficulties with preparing hot meals? (Definition: By preparing hot meals, we mean preparing ingredients, cooking, and serving food. [IWER: If another person prepares ingredients or if R can cook rice, but is not able to prepare side dishes, then mark (3).]
1. No, I don't have any difficulty
2. I have difficulty but can still do it.
3. Yes, I have difficulty and need help.
4. I can not do it.
DifficultyShopping(DB018) Because of health and memory problems, do you have any difficulties with shopping
for groceries? By shopping, we mean deciding what to buy and paying for it.
1. No, I don't have any difficulty
2. I have difficulty but can still do it.
3. Yes, I have difficulty and need help.
4. I can not do it.
DifficultyTakeMedications(DB020) Because of health and memory problems, do you have any difficulties with taking medications? By taking medications, we mean taking the right portion of medication right on time.
1. No, I don't have any difficulty
2. I have difficulty but can still do it.
3. Yes, I have difficulty and need help.
4. I can not do it.

Bodily pain

AnyBodyPains(DA041) Are you often troubled with any body pains?

1. Yes
2. No

WhatPartBodyPain(DA042) On what part of your body do you feel pain? Please list all parts of body you are currently feeling pain.

1. Head (Headache)
2. Shoulder
3. Arm
4. Wrist
5. Fingers
6. Chest
7. Stomach (Stomachache)
8. Back
9. Waist
10. Buttocks
11. Leg
12. Knees
13. Ankle
14. Toes
15. Neck

General health

SelfRatedHealth1(DA001) Next, I have some questions about your health. Would you say your health is excellent,
very good, good, fair, or poor?

[IWER：Interviewer should read all the following options]

1. Excellent
2. Very good
3. Good
4. Fair
5. Poor

SelfRatedHealth2(DA002) Next, I have some questions about your health. Would you say your health is very
good, good, fair, poor or very poor?

[IWER：Interviewer should read all the following options]

1. Very good
2. Good
3. Fair
4. Poor
5. Very poor

Vitality

SleepRestless(DC015) My sleep was restless.

1. Rarely or none of the time (<1 day)
2. Some or a little of the time (1-2 days)
3. Occasionally or a moderate amount of the time (3-4 days)
4. Most or all of the time (5-7 days)

CouldNotGetGoing(DC018) I could not get ”going.”

1. Rarely or none of the time (<1 day)
2. Some or a little of the time (1-2 days)
3. Occasionally or a moderate amount of the time (3-4 days)
4. Most or all of the time (5-7 days)

Social functioning

AnySocialActivities(DA056) Have you done any of these activities in the last month? (Code all that apply)

1. Interacted with friends
2. Played Ma-jong, played chess, played cards, or went to community club
3. Provided help to family, friends, or neighbors who do not live with you and who did not pay you for the help
4. Went to a sport, social, or other kind of club
5. Took part in a community-related organization
6. Done voluntary or charity work
7. Cared for a sick or disabled adult who does not live with you and who did not pay you for the help
8. Attended an educational or training course
9. Stock investment
10. Used the Internet
11. Other
12. None of these

FrequencyOfActivity(DA057) Frequency of activity in the last month

How often in the last month [did/have][you] [do voluntary or charity work/cared for a sick or disabled adult/provided help to family, friends or neighbors/attended an educational or training course/ Interacted with friends /go to a sport,social or other kind of club/taken part in a community-related organization]? Almost daily, almost every week, or not regularly?

(1) Almost daily

(2) Almost every week

(3) Not regularly

Role-emotional

TroubleConcentrate(DC010) I had trouble keeping my mind on what I was doing.

1. Rarely or none of the time (<1 day)
2. Some or a little of the time (1-2 days)
3. Occasionally or a moderate amount of the time (3-4 days)
4. Most or all of the time (5-7 days)

FeelHard(DC012) I felt everything I did was an effort.

1. Rarely or none of the time (<1 day)
2. Some or a little of the time (1-2 days)
3. Occasionally or a moderate amount of the time (3-4 days)
4. Most or all of the time (5-7 days)

Mental health

UnusuallyBothered(DC009) I was bothered by things that don’t usually bother me.

1. Rarely or none of the time (<1 day)
2. Some or a little of the time (1-2 days)
3. Occasionally or a moderate amount of the time (3-4 days)
4. Most or all of the time (5-7 days)

FeltDepressed(DC011) I felt depressed.

1. Rarely or none of the time (<1 day)
2. Some or a little of the time (1-2 days)
3. Occasionally or a moderate amount of the time (3-4 days)
4. Most or all of the time (5-7 days)

FeltHopeful(DC013) I felt hopeful about the future.

1. Rarely or none of the time (<1 day)
2. Some or a little of the time (1-2 days)
3. Occasionally or a moderate amount of the time (3-4 days)
4. Most or all of the time (5-7 days)

FeltFearful(DC014) I felt fearful.

1. Rarely or none of the time (<1 day)
2. Some or a little of the time (1-2 days)
3. Occasionally or a moderate amount of the time (3-4 days)
4. Most or all of the time (5-7 days)

Happy(DC016) I was happy.

1. Rarely or none of the time (<1 day)
2. Some or a little of the time (1-2 days)
3. Occasionally or a moderate amount of the time (3-4 days)
4. Most or all of the time (5-7 days)

FeltLonely(DC017) I felt lonely.

1. Rarely or none of the time (<1 day)
2. Some or a little of the time (1-2 days)
3. Occasionally or a moderate amount of the time (3-4 days)
4. Most or all of the time (5-7 days)
